# A computational model for designing energy behaviour change interventions

**DOI:** 10.1007/s11257-017-9199-9

**Published:** 2017-12-18

**Authors:** Nataliya Mogles, Julian Padget, Elizabeth Gabe-Thomas, Ian Walker, JeeHang Lee

**Affiliations:** 1 University of Bath, Claverton Down Rd, Bath, BA2 7AY UK; 2 University of Bristol, Senate House, Tyndall Ave, Bristol, BS8 1TH UK; 3 University of Plymouth, Drake Circus, Plymouth, PL4 8AA UK; 4 KAIST, 291 Daehak-ro, Guseong-dong, Yuseong-gu, Daejeon, South Korea

**Keywords:** Behaviour change, Energy consumption, Persuasive technology, Internal values, Energy literacy, Computational model, Simulation

## Abstract

The conflicting evidence in the literature on energy feedback as a driver for energy behaviour change has lead to the realization that it is a complex problem and that interventions must be proposed and evaluated in the context of a tangled web of individual and societal factors. We put forward an integrated agent-based computational model of energy consumption behaviour change interventions based on personal values and energy literacy, informed by research in persuasive technologies, environmental, educational and cognitive psychology, sociology, and energy education. Our objectives are: (i) to build a framework to accommodate a rich variety of models that might impact consumption decisions, (ii) to use the simulation as a means to evaluate persuasive technologies in-silico prior to deployment. The model novelty lies in its capacity to connect the determinants of energy related behaviour (values, energy literacy and social practices) and several generic design strategies proposed in the area of persuasive technologies within one framework. We validate the framework using survey data and personal value and energy consumption data extracted from a 2-year field study in Exeter, UK. The preliminary evaluation results demonstrate that the model can predict energy saving behaviour much better than a random model and can correctly estimate the effect of persuasive technologies. The model can be embedded into an adaptive decision-making system for energy behaviour change.

## Introduction

As a result of environmental and economic concerns arising from the use of energy, energy consumption issues have received enormous attention in recent years both at international and national levels. One of the foci of research on and aspirations for the reduction of domestic energy consumption lies now in inducing individual human behaviour change with the help of technological solutions: smart meters or ambient displays (Darby 2001) that are capable of providing continuous daily feedback on household energy consumption.

Some research findings (Abrahamse et al. 2005) suggest that continuous energy feedback might be an effective driver for energy behaviour change. For example, Barbu et al. (2013) suggest energy feedback provided to users via smart meters could save 5–15% of energy costs. Similarly, Darby (2010), Hargreaves et al. (2010) and Vine et al. (2013) suggest that energy feedback through advanced in-home displays (IHD) could help to save up to 20% of energy costs. Although some reported savings seem quite moderate, they indicate that intelligent energy feedback that offers different feedback options might be an effective means to achieve energy reduction targets (Fischer 2008). However, the supporting technological solutions suffer from multiple drawbacks (Buchanan et al. 2015; Fitzpatrick and Smith 2009; Hargreaves et al. 2010): the reported energy savings in the studies above are very variable and the majority of the interventions are not reproducible because “interventions are not systemically designed, documented, implemented, and evaluated” (Karatasou et al. 2014). This variability in systematisation of energy behaviour change interventions is due to methodological and theoretical limitations, which have been reported in the energy literature and can be summarised as follows (Buchanan et al. 2014, 2015): (i) disinterested users, (ii) failure to address users’ personal motivations and needs embedded in daily routines and social practices, (iii) information comprehension issues caused by abstract numerical information in kWh and financial costs (low energy literacy), and (iv) inattention to users’ personal characteristics. These findings give a clear indication that users need something more to engage them than plain energy feedback in power or monetary terms. Indeed, energy consumption behaviour is a very complex phenomenon, intermingling social and cultural aspects, which requires a more rigorous and a more holistic approach. Different attempts have been made to improve plain numeric energy feedback. For example, in the study of McCalley and Midden (2002) the effect of goal setting and social orientation was explored in a laboratory experiment on energy feedback, in Midden and Ham (2008) an effect of social energy feedback was demonstrated, again in laboratory conditions. Abrahamse et al. (2007) applied a combination of tailored information, goal setting and tailored feedback in their field experiment on energy feedback. In Handgraaf et al. (2013) a field study investigated how monetary and social rewards incorporated in energy feedback influence users, while a large scale field experiment (Schultz et al. 2015), social influence strategy was tested against plain numeric and monetary feedback.

Energy behaviour change constitutes a substantial body of research within the Human–Computer Interaction (HCI) field and contributes to the research in sustainable HCI (DiSalvo et al. 2010) and eco-feedback technologies (Froehlich et al. 2010). Eco-feedback technology provides feedback on individual or group behaviours with an aim of reducing environmental impact. This area of research is part of the persuasive technologies subfield of HCI and comprises about half of all persuasive applications (DiSalvo et al. 2010). Rapid development in mobile applications and social media creates favourable conditions for the implementation and realisation of persuasive applications. There is some evidence reported in the literature that persuasive technologies have in general a positive effect on users’ behaviour (Hamari et al. 2014), although their impact depends on how effectively they incorporate influencing strategies from the social sciences. Some interventions based on persuasive technologies in that energy consumption domain have had variable success due to the lack of formalised design approaches that implement findings from theoretical research in environmental psychology and other social sciences (Froehlich et al. 2010; Petkov et al. 2012; Dourish 2010). “Design decisions in persuasive applications are often taken intuitively, rather than being theoretically determined” and “provide the same feedback to different people” while different users might be susceptible to different influences (Petkov et al. 2012). For this reason research on personalisation within persuasive technologies has gained a lot of interest in recent years (see for example Berkovsky et al. 2012; Kaptein et al. 2012; Orji 2014).

There is a need for a more personalised and an holistic approach towards the design of persuasive technologies in energy conservation by taking into consideration multiple individual and social determinants (Froehlich et al. 2010; Dourish 2010; Mozo-Reyes et al. 2016). There has been work on formalising persuasive strategies, e.g. a Model for Adaptive Persuasion (MAP) (Kang et al. 2015), which is based on the Elaboration Likelihood Model (Cacioppo et al. 1986) and was embedded into a virtual agent that provides support for healthy lifestyle behaviour. Or the work of Thieme et al. (2012) on a social persuasive system based on the Theory of Planned Behaviour (Ajzen 2011). Also in the work of Mozo-Reyes et al. (2016) and Petkov et al. (2012), findings from environmental psychology and Value-Belief-Norm theory (Stern 2000) were addressed. However, to the authors’ knowledge, there are neither any energy behaviour change computational models extant that integrate multiple theories of energy behaviour change determinants in a systematic and structured way, nor any simulation of intervention mechanisms based on these determinants. Some research has been done in the Artificial Intelligence (AI) domain on computational modelling of human agents’ determinants of environmentally-friendly behaviour, such as internal values, personal norms and energy awareness (Sánchez-Maroño et al. 2013, 2014) with the aim of facilitating practical applications in the area of digital solutions towards intelligent energy behaviour change interventions. However, the approach is not aimed at the usage of persuasive technologies at an individual level, but is related to managerial and policy making decision processes. In the work of Hammer et al. (2015), a computational User Trust Model for tailored automatic control of smart energy systems is presented, based on Bayesian networks, though the focus of this model lies on the design of automatic control systems and not on persuasive technology and user behaviour change.

In consequence, and for the motivations set out earlier, we propose a computational agent-based model that integrates multiple theories underlying energy consumption behaviour and simulates the effect of different types of energy feedback. The model combines research findings from persuasive technologies, environmental, educational and cognitive psychology, sociology and energy education and provides a more holistic and transparent approach towards intelligent behaviour change interventions, as it is based on multiple rather than one theory of energy consumption behaviour and on a deterministic modelling approach. Further, the model does not only simulate the mechanisms behind energy consumption, but also the dynamic effect of behaviour change interventions. The main focus of interventions within the model lies on internal human values (Schwartz 1992; Stern 2000; Steg et al. 2014b) and energy literacy (Cotton et al. 2016) components, as they have gained most attention in recent years in the research fields of behaviour change and energy consumption. The model we propose offers the following properties:

1. An explicit and transparent framework for the design of energy behaviour change interventions
2. Means to explore the mechanisms and dynamics behind behaviour change interventions and how they work at a cognitive level
3. A model that can be embedded into an intelligent agent to reason about behaviour change strategies and to apply the most appropriate given the user context
4. A component of a persuasive system, either at a design or an implementation stage, in which the agent-based model forms the basis for tailored interventions that offer the potential to improve user engagement and enrich the user experience.

The proposed model incorporates Fogg’s model of persuasive design (Fogg 2009a) and connects it with recent findings on internal values from environmental psychology (Stern 2000; Steg et al. 2014b), and on energy literacy from energy education research (Cotton et al. 2016). We performed four model evaluation studies in order to investigate the following aspects of model performance: (i) the persuasive potential of personalisation based on internal values; (ii) the most influential model inputs; (iii) if the model makes correct predictions of energy consumption behaviour states; (iv) if the model makes correct predictions regarding the effect of such persuasive strategies as *tailoring*, *reduction* and *tunneling* proposed in Oinas-Kukkonen and Harjumaa (2008). In order to answer these research questions, we used psychological data on energy-related behaviour determinants, such as energy literacy, perceived barriers, personal values, success expectancy, and electricity consumption data obtained from 20 households in Exeter, UK, collected between January 2014 and April 2016, and survey data on personal values and energy feedback preferences from 30 individuals in Bath, UK, collected in July 2015. We used sensitivity analysis in order to answer research question (ii) described above.

The preliminary model evaluation demonstrated that: (i) energy information preferences of individuals were partially associated with their internal values, which implies tailoring interventions according to personal values as the model suggests; (ii) the model, with inputs obtained from real data, predicted the energy-saving behaviour of households much better than a model with random inputs; (iii) the model made good predictions of the effect of digital interventions, measured as a change of energy-saving behaviour over time.

The model can guide design of persuasive technologies for energy consumption behaviour change and can provide a preliminary simulation-based evaluation of this behaviour prior to the deployment of these technologies. It can also provide estimations of persuasive power of such technologies for specific users.

The rest of this paper is organised as follows: Sect. 2 describes the modelling framework in the area of Ambient Intelligence selected for the research presented here and explores the background literature on generic behaviour change strategies, energy-related behaviour determinants; Sect. 3 presents a conceptual model for behaviour change interventions dynamics. In Sect. 4 the formalisation of the conceptual model is specified; Sect. 5 describes four studies performed for the purposes of the model evaluation; In Sect. 6, model simulations with different input scenarios are described and finally Sect. 7 concludes the work.

## Theoretical background

This section describes the modelling framework and the theoretical foundations of behaviour change frameworks.

### Modelling framework

**Fig. 1:**
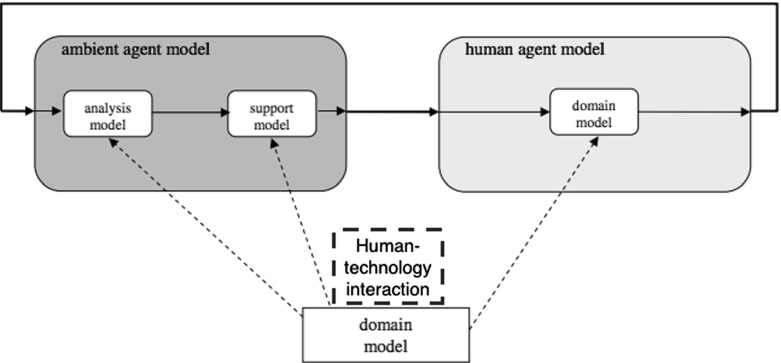
Ambient agent framework for design of human support systems. (Reproduced with permission from Bosse et al. 2011)

There are numerous cognitive architectures proposed in the area of Cognitive Science, the most heavily cited of which are ACT-R (Anderson and Lebiere 2014), SOAR (Laird et al. 1987) and COGENT (Cooper and Fox 1998). Potentially these architectures can be adopted for user modelling within the persuasive technologies domain. However, they consist of several interrelated modules and are not so flexible in terms of a specification of additional non-deliberative cognitive processes, because they were developed for other purposes. The ambient agent modelling framework (Bosse et al. 2011) was specifically developed for applications in HCI and Ambient Intelligence. We adopt this framework because we believe it is the most suitable for modelling human agents in the context of persuasive technologies. Another modelling framework developed within the area of Ambient Intelligence (Kaptein et al. 2010) relies on multiple theories of behaviour change, although it does not focus on the intelligent tailoring mechanisms within persuasive technologies. The architecture presented by Bosse et al. (2011) aims to provide software agents with a rich knowledge of humans and the environment where human agents operate, a human-like understanding of human functioning and hence effective tailoring of persuasion strategies. Within this framework, the knowledge about the user is expected to be represented by being integrated within software agents models of humans and is referred to in the framework as a ‘domain model’. The domain model is informal knowledge about humans and their environments, which acts as a basis for a formal representation that can be embedded within a formal model of a human agent (see Fig. 1). Solid arrows represent information flows and dotted arrows represent derivation paths. By incorporating domain models within an agent model, the Ambient Intelligence agent gets an understanding of the processes of its surrounding environment, which is the basis for knowledgeable intelligent behaviour.

The framework described in Bosse et al. (2011) proposes the integration of domain knowledge in three ways:

1. **Domain model** using a domain model directly as the (human) agent model (right hand side of Fig. 1). In this case, a domain model that describes human processes and behaviour is used directly as an agent model, in order to simulate human behaviour.
2. **Analysis model** to perform analysis of the human actor’s states and processes, by reasoning based on observations (possibly using specific sensors) in combination with the domain model (left hand side of Fig. 1).
3. **Support model** to provide support for human actions by reasoning based on the domain model and making changes in the environment that aim at influencing human cognitive states and actions.

In the work presented here, we focus on the human agent model (right hand box in Fig. 1) since we model human cognitive processes and behaviour rather than an artificial software agent supporting a human. Further, within the *domain model* we include not only the knowledge of human cognitive processes, but also that of the dynamics of specific kinds of human–technology interaction.

### Behaviour change frameworks

Behaviour change frameworks can be roughly divided into two kinds according to the research areas where they are developed: (i) behaviour change approaches in the health domain, and (ii) approaches in the domain of HCI and persuasive technologies. Frameworks developed in the first group do utilise digital technologies while delivering interventions, but their focus is broader and includes non-digital interventions as well. Approaches from the second group are explicitly focused and rely on digital technologies for persuasive communications.

In the HCI and persuasive technologies domain, Fogg (2009a) proposes a simplified model of behaviour change supported by technology, and based on various psychological theories. Fogg’s model states that for a desirable behaviour to take place, three elements are needed: (i) motivation, (ii) ability/resources, and (iii) trigger/cue, all of which should occur at the same time. The role of technology here is to provide a specific cue for the behaviour that is of interest, although a key question that remains, is what form that cue should take: it might be information, a motivational message, encouragement, reward, behaviour prompts or reminders or any other persuasive communication method. Fogg’s model is based on earlier work from marketing research which stressed motivation, opportunity and ability (MOA model) (Maclnnis and Jaworski 1989). Fogg and Hreha (2010) later proposes a behaviour change intervention framework based on seven strategies that matches target behaviours with solutions, however, it does not explain the mechanisms of working of the proposed interventions. The strategies proposed by Fogg are *reduction, tunnelling, tailoring, suggestion, self-monitoring, surveillance, conditioning*.

In Oinas-Kukkonen and Harjumaa (2008) a systematic framework for the design of persuasive systems is proposed by Oinas-Kukkonen. The framework consists of 28 persuasive system design principles. It builds upon earlier versions of Fogg’s behaviour change strategies framework (Fogg 2009b) and extends it with additional strategies. The first seven principles listed in the work of Oinas-Kukkonen are related to the primary task support and are the following: *reduction, tunnelling, tailoring, personalisation, self-monitoring, simulation, rehearsal*. The frameworks presented in Oinas-Kukkonen and Harjumaa (2008) and Fogg (2009b) appear to be the most influential in support of systematic behaviour change interventions strategies, however it is not clear how each of these principles or strategies can be implemented in a particular user context. An explicit user model is needed in order to implement these strategies and to get more insights into the mechanisms of their working. It provides us with a direction to develop a more explicit computational model of a human user and to incorporate working mechanisms of different strategies within this model. Consequently, in the work presented here, we seek to obtain a partial view inside the “black box” of a user to provide more detailed directions and guidance for persuasive systems design, in particular we investigate what can be the focus of this technological trigger/cue component.

In the field of health-related behaviour change, Michie et al. (2011) propose a model based on the findings of behavioural theorists and of principles found in US criminal law. It comprises three factors: capability, opportunity and motivation. Michi et al. propose behaviour change strategies using this model, but the model representation is too generic and lacks sufficient concrete details to explain the working mechanisms of the interventions. Furthermore, the model does not account for the dynamics of behaviour change given particular interventions. Prochaska and DiClemente (1984) propose a trans-theoretical model for behaviour change given the dynamics of behaviour change stages and transitions from one stage to another. Once again, the model is not able to account for the working of particular interventions and does not contain any components that would allow for the delivery of persuasive technologies.

Some findings suggest that theory-based behaviour change interventions are more effective than interventions which do not explicitly mention or incorporate a behaviour change theory (Taylor et al. 2012), others on the contrary state that theory-based interventions are *not* more effective than non-theory based ones (Azar et al. 2013). Despite these controversial results in the literature regarding the necessity or not for a supporting theory, it is not contested that theories can be very useful in the design of automated behaviour change systems, since they offer intervention frameworks and transparency guidelines for the evaluation of these systems (Hekler et al. 2013). The most influential behaviour change intervention frameworks and models are theory based (Prochaska and DiClemente 1984; Fogg 2009b; Michie et al. 2011) and we believe that it is essential to incorporate theories into behaviour change models and interventions. It is also suggested in Hekler et al. (2013) to choose theory-based conceptual frameworks, rather than abstract meta-models, to inform the design of behaviour change systems, because they are more specific than meta-models and at the same time more generalisable than pure empirical studies.

Given the existing persuasive design principles and lack of explicit, less abstract behaviour change user models, in our work we are aiming to enrich persuasive strategies proposed in literature on persuasive technology with an explicit formal computational user model based on the most influential behaviour change theories mentioned in an energy consumption domain. The proposed model is able to demonstrate dynamic aspects of persuasive technologies and to simulate their effect over time.

We selected to focus on the first three principles of Oinas-Kukkonen framework for building persuasive technologies—*reduction, tunnelling, tailoring*—and to implement them within a computational user model for persuasive technologies. The proposed model is based on Fogg’s basic user model since the core of the model [MOA model (Maclnnis and Jaworski 1989)] is widely appreciated by researchers (Kaptein et al. 2010). In the current work, the Fogg’s model is extended with theories on energy consumption from environmental, social and educational psychology and social sciences. It is also formalised and implemented in the MATLAB software environment which allows for simulations of different persuasion strategies and scenarios.

## Conceptual model

**Fig. 2:**
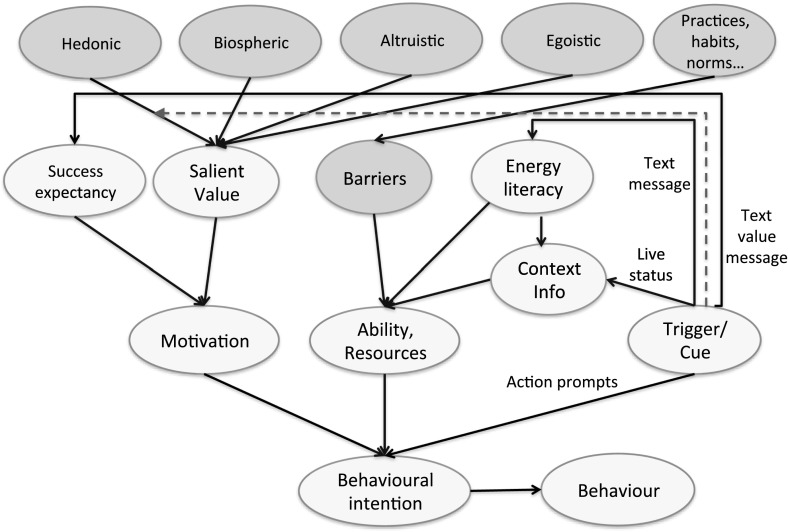
Cognitive agent-based model of energy behaviour change interventions mechanisms for persuasive technologies. The grey ovals represent static determinants of behaviour not susceptible to persuasion. Solid arrows represent connections between the components and directions of influence, a dotted blue arrow represents indirect influences through weights change. (Color figure online)

This section describes the theoretical foundations of the proposed energy consumption behaviour model and the structure of the model. The proposed energy behaviour change model we believe represents the main psychological and social components that should be addressed during human–computer interaction process. The computational model is depicted in Fig. 2.

The model is a result of an integration of three important frameworks: Fogg’s model of persuasive technologies (Fogg 2009a) (the bottom of Fig. 2), value-based (Steg et al. 2011, 2014a) and goal framing (Lindenberg 2001) approaches to sustainability behaviour from environmental psychology and the energy literacy concept (Cotton et al. 2016) from educational psychology (the top of Fig. 2), as they are the most influential in the field of energy consumption. The grey ovals represent static determinants of behaviour, which cannot be directly influenced by persuasive technologies and interventions at an individual level. The peach coloured elements denote dynamic components, which are susceptible to change by means of persuasive technologies. This classification into changeable and unchangeable behaviour determinants within the model is based on the literature in social sciences. The intermediate layer of the model, which is one of the main contributions of the paper, consists of the components labelled *success expectancy*, *salient value*, *barriers*, *energy literacy*, *context information*. These serve to integrate Fogg’s approach and the approaches from environmental psychology into a feedback mechanism to connect with behaviour change. Suitable theories were selected to match these two approaches from different research fields according to the principles of complementarity and overlap. Solid arrows represent connections between the components and directions of influence, a dotted arrow such as that from *trigger/cue* to the internal value connections, represents indirect influences through internal values activation effects by means of the changed connection weights.

**Motivation** From the persuasive technologies model perspective, for a behaviour to be changed three elements are needed and they should happen at the same time: *motivation*, *ability/resources* and *trigger/cue*. In order to influence a person’s *motivation*, one should have a clear picture of motivational determinants and motivation formation. In the psychology literature, different motivational theories are mentioned: theories focused on expectancies for success (self-efficacy and control theories), theories focused on task value (intrinsic motivation, self-determination, flow, interest, and goals), theories that integrate expectancies and values (attribution theory, the expectancy-value models, self-worth theory), and theories integrating motivation and cognition (social cognitive theories of self-regulation and motivation, theories of motivation and volition) (Eccles and Wigfield 2002).

According to the expectancy value motivation theory (Eccles and Wigfield 2002), two main components contribute to motivation: the value that you attribute to a particular behaviour and the expectancy of success of this behaviour’s outcomes. In our model, we propose to integrate Fogg’s motivation component with value (Schwartz 1992) and goal framing (Lindenberg 2001) approaches, aimed at environmental behaviour, using expectancy value motivation theory as a connecting component. We have extended the motivation component of Fogg’s model with findings from educational and environmental psychology. In the proposed model, a salient internal value, which is activated at a particular moment, is operationalised as the *salient value* component in Fig. 2 and is a function of the four environmental values identified in the literature (Steg et al. 2011, 2014a): *hedonic*, *biospheric*, *altruistic* and *egoistic*.

In our model, goal framing theory based on internal values and expectancy value theory feed into the motivation component of Fogg’s model.

**Internal Values** The findings from the environmental psychology literature postulate that environmental behaviour is highly correlated with some internal values: altruistic, biospheric, egoistic and hedonic (Steg et al. 2014a). Internal values are “desirable goals, varying in importance, that serve as guiding principles in people’s lives” (Schwartz 1992). Values are considered to be relatively stable over time. Values are conceptually different from the goals and attitudes: they reflect which goals people find most important in life in general, while goals reflect what motivates people in a given situation, which not only depends on their values but also on situational cues (Steg et al. 2014b).

**Goal Framing** Goal-framing theory (Lindenberg 2001) acknowledges that behaviours result from multiple motivations. Three general goals, or motivations, are distinguished according to this theory: a hedonic goal-frame, a gain goal frame and a normative goal frame. The goal that is triggered becomes focal and thus has the greatest influence on motivational and cognitive processes and give them temporarily greater weight than the other two goals. The term ‘triggered’ indicates that the goal frames are not chosen, but are subject to automatic priming effects (Lindenberg and Steg 2013). Energy feedback delivered by persuasive technologies via an IHD, a mobile phone or a computer can serve as an environmental cue that is able to frame energy information according to users’ values.

It is suggested that goal framing theory can be naturally integrated with the value approach towards environmental behaviour (Steg et al. 2014b). Within the motivational context, an expectancy value motivational theory (Wigfield et al. 2000) forms a logical connection between personal values and motivations.

**Energy Literacy** Since energy consumption directly involves a broad range of materials and technology, e.g. transportation, appliances and infrastructure either for thermal or social comfort, hygiene, aesthetics, it becomes necessary that an individual have a reasonably accurate representation of how all these energy using technologies operate, and what impact they have on environment and society. In other words, energy consumption behaviour change can occur only if people understand how this technology works. However, knowing how to operate an appliance that one is using on a daily basis, such as an electric kettle or a boiler, is not enough to induce more pro-environmental energy consumption behaviour. Some scientific knowledge of energy and general understanding of energy systems and how energy using technology operates is very important here. This in-depth understanding of energy consumption is being referred in pedagogical, educational and environmental policy literature as *energy literacy* (Cotton et al. 2016). Energy literacy is “a broad term encompassing content knowledge as well as a citizenship understanding of energy that includes affective and behavioural aspects” (DeWaters and Powers 2013). Cognitive (knowledge, in-depth understanding), affective (attitudes, values), and behavioural (social practices) modules can be distinguished within a complex concept of energy literacy. In our model, under energy literacy, we assume only the cognitive component of energy literacy defined in DeWaters and Powers (2013) since the other two components are integrated within *barriers* (social practices, habits) and *motivation* elements of the proposed model (see Fig. 2).

**Social Practices** Social practice theorists emphasise the role of social environment (social norms, social practices, materials, infrastructure) and agent-environment interaction and development (Bourdieu 1977; Turner 1986; Shove 2014). They state that the practice itself should become the core unit of analysis, rather than the individuals who perform them. In the contemporary context of energy consumption or sustainability behaviour, practices or everyday routines, habits (Aarts and Dijksterhuis 2000) function as *barriers* for behaviour change interventions at an individual level (Hargreaves 2011).

**Situation Awareness** The idea of inducing behaviour change interventions with the help of smart meters (Darby 2010) is similar to the idea of providing continuous dynamic information on the context of energy related operations and behaviour within a household, which is in line with the psychological concept of *situation awareness* (Endsley 1995). This concept is widely applied in the design of safety critical systems and in providing task-related support for operators in dynamic environments. Undoubtedly, in the context of domestic energy use having a situational awareness of what is going on with energy within a person’s home might be very helpful for energy consumption decisions, though this component of energy feedback is not the most important one. People have quite limited knowledge of energy operations and are less motivated to undertake several actions in this direction, in comparison to, for example an operator of a nuclear power plant. The situational awareness component in our model is called *context information* to avoid a contamination by the *situation awareness* term coming from a different area.

**Abilities** The concepts of energy literacy, social practices/habits and context information (situation awareness) naturally match the concept of *ability/resources* from Fogg’s model. The * abilities/resources* component in the proposed model is a function of perceived *barriers* related to an energy consumption behaviour change, knowledge of how energy is being used in the *context* of a person’s household and *energy literacy*—relevant knowledge on how to overcome these barriers. Barriers represent socio-cultural environment and infrastructure wherein a person is situated, e.g. social practices, norms, expectations, stable habits formed as a result of these repetitive practices. It is difficult to change these perceived barriers during individual interventions, but one can provide useful tips and concrete prompts how to overcome them.

**Behavioural Cue** A useful property of digital technologies and intelligent support systems is that you have always a behavioural *trigger* or a *cue*, which is an interactive system itself. The *trigger/cue* from Fogg’s model can address different aspects of energy behaviour components. For example, it can address external behaviour by communicating direct messages and prompts what to do (e.g. “Write a note reminding yourself to turn off the heating when you leave home. Or reset your heating timer”).

Apart from motivational messages, a persuasive system can deliver a direct energy feedback similar to the information provided by contemporary In-Home-Displays (IHD) of smart meters. This energy feedback is a way of improving situation awareness. It represents the dynamics of energy usage in a particular household and is operationalised as energy context information within the model. It is important to note that within the situation awareness framework (Endsley 1995) the ‘mental model’ component is an important factor for the interpretation of observations and belief formation. This ‘mental model’ is represented as energy literacy in the proposed integrated model. Energy literacy has influence on both ability/resources and perception of the context information (direct energy feedback cue). Further, a cue component can activate dominant values associated with environmental behaviour by changing the connection weights between the values and a salient value and contributing to an increased motivation component.

According to several psychological models of behaviour change, a precursor of each behaviour is behavioural intention (Gollwitzer 1999; Ajzen and Madden 1986). It is unlikely that a cue presented to a person will immediately induce an action, or actual behaviour. Cues are being processed in a cognitive information processing system first, either at a conscious or unconscious level, before they are translated into behaviour. For this reason we decided to include a *behavioural intention* component between behaviour and motivation, and ability and cue components of Fogg’s model.

As it can be seen in Fig. 2, a cue/trigger has both direct and indirect effect on the target behaviour: via a direct link to the behaviour of interest and via four other links—to context information, energy literacy, value connections and success expectancy. We hypothesise that tailored information within the cue can raise both energy literacy and success expectancy and can produce some long term effect on these components by affecting human memory.

We assume in our model that each cue/trigger contains an educational element that influences the energy literacy aspect and helps to diminish the influence of barriers. The cue can also frame information messages according to the four internal values: hedonic, biospheric, altruistic, egoistic. This allows for an activation of motivations relevant to the energy usage behaviour. Within the proposed conceptual model, all above described concepts related to energy behaviour and behaviour change interventions are integrated in a form of four hierarchical layers with the help of deterministic causal relations.

## Model formalisation

The model described in the previous section integrates multiple theories of energy behaviour determinants and behaviour change interventions (see Fig. 2). It has been formalised and implemented in MATLAB. All model values and parameters are expressed within a [0,1] interval and all weights are normalised so they sum to 1. All formulas used in the calculation of behaviour change variables are weighted sums of the influencing components, except for success expectancy and energy literacy which are dynamic: a variable value at a next time point depends also on the variable’s value at a previous time point. For the formalisation of these dynamic relations we followed a temporal-causal network modelling approach for cognitive modelling as described in Treur (2016). The temporal-causal network modelling approach is an attempt to approximate relations between cognitive phenomena with the help of mathematical formulas given theoretical knowledge obtained from psychology literature.

There are eight inputs to the model: (i) four internal values: hedonic, biospheric, altruistic, egoistic (ii) success expectancy (iii) barriers (iv) energy literacy and (v) trigger/cue. A cue or trigger is presented at each step of the model simulation, while the output of the model is a behavioural intention that may be translated into actual behaviour subject to a particular threshold.

**Cue** A cue is complex information determined by a behaviour change intervention. The value of cue $V_{cue}$ is an input vector consisting of 6 elements that represent the following components of a cue: (i) value neutral energy information $V_{cuec}$, (ii) hedonic $V_h$, (iii) egoistic $V_e$, (iv) altruistic $V_a$, (v) biospheric $V_b$ value framed energy information and (vi) action prompts component $V_{cue\_action}$:

1 $$\begin{aligned} V_{cue} =(V_{cuec}, V_h, V_e, V_b, V_a, V_{cue\_action}) \end{aligned}$$

**Salient value** The salient value $V_{val}$ represents a person’s situational evaluation of pro-environmental behaviour. The salient value is a weighted average of four internal values associated with pro-environmental behaviour: hedonic $V_h$, egoistic $V_e$, biospheric $V_b$ and altruistic $V_a$. The weights $w_h$, $w_e$, $w_b$, $w_a$ are the connections from the four respective values with the current salient value. The value of $V_{val}$ changes depending on the degree of activation of different internal values triggered by situational cues. Within the connections $w_h$, $w_e$, $w_b$, $w_a$, a connection set is activated, being either one that is not tailored to a dominant internal value (i.e. all value connections are equal), or or one tailored to a dominant value (i.e. where one value is much higher than the other three), such as when messages are tailored according to personal values:

2 $$\begin{aligned} V_{val} = \sum _{i\in \{h,e,b,a\}} V_i*w_i \end{aligned}$$

For example, if no cue is presented, each weight is equal to 0.25 and if a cue or trigger is presented, the assumption is that the cue is designed to trigger a particular internal value in which case the weight of that value will be equal to 0.9 and the weights of the other three values will be equal to 0.03. All weights in an expression of a weighted sum always sum to 1.

**Success expectancy** In the temporal relation for success expectancy $V_{suc}$, the previous success expectancy is taken into account, as well as an information component $V_{cuec}$ extracted from the behaviour change intervention cue input vector $V_{cue}$ (see Eq. 1). Here $\gamma$ is an information contribution parameter: it determines the amount of increase of success expectancy based on the energy information provided by the cue. The initial value of $\gamma$ was selected arbitrarily with trial and error based on the assumption that the value increase per time step is smooth and gradual and should not exhibit high jumps according to the psychology literature. The increase is proportional to the difference in time, with proportion factor $\gamma * \varDelta t$ as described in Treur (2016). The function *Min*(*x*) here and in Eq. 5 is defined as the minimum of its argument and 1, so the result is in [0, 1]:

3 $$\begin{aligned} V_{suc}(t+\varDelta t) = Min(V_{suc}(t) + \gamma * V_{cuec}(t) * (1-V_{suc}(t)) * \varDelta t) \end{aligned}$$

**Motivation** A person’s motivation is a weighted average of salient value $V_{val}$ and success expectancy of energy conservation behaviour $V_{suc}$. Weights $w_{val}$, $w_{suc}$ are the connections between salient value and success expectancy respectively and motivation.

4 $$\begin{aligned} V_{mot}(t) = V_{val}(t) * w_{val} + V_{suc}(t) * w_{suc} \end{aligned}$$

There are two weights in this expression and since the contribution of success expectancy and silent value is assumed to be equal given the theoretical description of the expectancy-value theory, each of them is equal to 0.5 so that they sum to 1.

**Energy literacy** By Energy literacy we mean general energy literacy, or energy-related beliefs. Here we assume that the energy literacy component of the model is dynamic because the information provided by the trigger/cue ought to increase the knowledge an individual has about energy. If no trigger is presented to a user, then literacy remains unchanged. In this temporal relation for energy literacy $V_{lit}$, the previous energy literacy is taken into account, as well as an information component $V_{cuec}$ extracted from a behaviour change intervention cue input vector $V_{cue}$ (see Eq. 1). Here $\eta$ is an information contribution parameter, which determines the increase in energy literacy based on the energy information provided by the cue. Similar to the initialisation of the information contribution parameter $\gamma$ for Success expectancy, its initial value is selected arbitrarily with trial and error based on the assumption that the value increase of Energy literacy per time step should change quite slowly according to the psychology literature. The increase is proportional to the difference in time, with proportion factor $\eta * \varDelta t$, again as described in Treur (2016):

5 $$\begin{aligned} V_{lit}(t+\varDelta t) = Min(V_{lit}(t) + \eta * V_{cuec}(t) * (1-V_{lit}(t)) * \varDelta t) \end{aligned}$$

**Context information** Context information $V_{context}$ is related to the specific energy consumption information in a particular household. A smart meter with an energy consumption In-Home Display (IHD), or a mobile energy feedback application are examples of digital channels that provide this type of information. Context information is a weighted average of the energy literacy $V_{lit}$ and the information component $V_{cuec}$ extracted from a behaviour change intervention cue input vector $V_{cue}$ (see Eq. 1). Weights $w_{litc}, w_{cuec}$ are the connections between energy literacy and an informational component of cue, respectively, and context information that define how a person will process the energy behaviour change cue.

6 $$\begin{aligned} V_{context}(t) = V_{lit}(t) * w_{litc} +V_{cuec}(t) * w_{cuec} \end{aligned}$$

We assume that the level of energy literacy is much more influential here than information contained in the cue because one cannot process information about consumption without basic prior knowledge of how energy is measured. Hence, we assigned the value of 0.8 to $w_{litc}$ and the value of 0.2 to $V_{cuec}$ .

**Ability** Ability $V_{ability}$ defines the ability of a person to perform the new behaviour. Ability $V_{ability}$ is a weighted average of Barriers $V_{bar}$ and Energy literacy $V_{lit}$ and Context information $V_{context}$. Weights $w_{bar}, w_{lit}, w_{context}$ are the connections between Barriers $V_{bar}$, Energy literacy $V_{lit}$, Context information $V_{context}$ and Ability $V_{ability}$.

7 $$\begin{aligned} V_{ability}(t) = V_{bar} * w_{bar} +V_{lit}(t) * w_{lit} + V_{context}(t) * w_{context} \end{aligned}$$

There are three weights in this expression: $w_{bar}, w_{lit}, w_{context}$. The contribution of barriers, energy literacy and context information to ability are initially assumed to be equal and all are given the value of 0.33, so that they sum to 1.

**Behavioural intention/Behaviour** Behavioural intention is a precursor of actual behaviour according to the Theory of Planned Behaviour (Ajzen 2011). Note that in the current version of the model we do not need to differentiate between behavioural intention and behaviour because of the model’s level of analysis and the purpose the model serves. Here, behavioural intention $V_{beh}$ is a weighted average of motivation $V_{mot}$ and ability $V_{ability}$ and an action prompt component $V_{cue\_action}$ extracted from a behaviour change intervention cue input vector $V_{cue}$ (see Eq. 1). Weights $w_{mot}$, $w_{lit}$, $w_{ability}$, $w_{cueb}$ are the connections between motivation $V_{mot}$, ability $V_{ability}$, and the action prompt component $V_{cue\_action}$ of cue delivered by behaviour change interventions:

8 $$\begin{aligned} V_{beh}(t) = V_{mot}(t) * w_{mot} +V_{ability}(t) * w_{ability} + V_{cue\_action}(t) * w_{cueb} \end{aligned}$$

AlThough the description of Fogg’s model implies that all three constituents—motivation, ability and cue/trigger—have equal contributions to behaviour, our assumption is that a trigger has lower contribution compared to motivation and ability. Consequently, we assigned the value of 0.2 to weight $V_{cue\_action}$ and the value of 0.4 to $V_{mot}$ and $V_{ability}$.

All initial model parameters are listed in Table  1.

**Table 1:** Parameter values used in the model

Parameter	Value	Parameter	Value
$w_h$	0.25	$w_{context}$	0.33
$w_e$	0.25	$w_{bar}$	0.33
$w_a$	0.25	$w_{litc}$	0.8
$w_b$	0.25	$w_{cuec}$	0.2
$w_p$	0.9	$w_{mot}$	0.4
$w_w$	0.03	$w_{suc}$	0.5
$w_val$	0.5	$w_{lit}$	0.33
$w_{ability}$	0.4	$w_{cueb}$	0.2
$\eta$	0.005	$\gamma$	0.01

## Model evaluation

During the model evaluation we were investigating the following research questions: (i) What is the persuasive potential of personalisation based on internal values? The primary hypothesis to investigate here is whether the effect on behaviour change is better if interventions (messages) are adapted to user values; (ii) Which insights can we get from model sensitivity analysis with respect to an identification of the most influential model inputs and parameters? (iii) Does the model make correct predictions regarding energy consumption behaviour states? In order to be able to inform the design of persuasive technologies, a model should make correct predictions of users’ behaviour. We define behaviour as a set of different domestic energy behaviours, such as electricity usage and usage of heating and hot water. An operating definition of behaviour (for this paper) is electricity consumption as a proxy for all electricity-usage related behaviours, such as usage of electric appliances; (iv) Can the model simulate the effect of persuasive technologies? Here we investigate if the proposed model is able to predict the dynamics of energy saving behaviour as a function of persuasive technologies. We performed four model evaluation studies to answer these questions, which we now detail.

### Study 1: Persuasive potential of personalisation based on internal values

The aim of this study is to evaluate the relations between personal values and a value-framed energy information cue. Here value-framed and neutral energy feedback messages are presented to participants on paper to serve as cues. Our hypothesis is that the appeal and persuasive power of value-framed messages is highly correlated with respective values.

**Procedure** 30 participants were recruited via a local community center, a local café and from university academic staff. Mean age $=$ 34.3, median $=$ 32, minimal age $=$ 18, maximal age $=$ 60; 16 males, 14 females; 14 from working class with low income, 15 from middle class, 1 unknown. There was a prize draw for the half of the participants from lower social classes in order to motivate them to complete the surveys: two supermarket vouchers £10 each. The rest of the participants were not informed of any prize draw and did not receive any financial reward. Participants were asked to sign a consent form and to fill in an environmental values questionnaire (adapted from Groot and Steg 2008; Steg et al. 2014a), along with some demographic information and a survey on the expression of preferences for six short electrical energy information messages that were developed for this study. Both the questionnaire and the six messages responses were given on a 5-point Likert scale: “Below you will see 6 energy usage messages. How appealing are these messages to you? Choose a number between 1—Not appealing to 5—Very appealing”. Four of the six messages were framed according to the four values that are correlated with environmental behaviour: hedonic, egoistic, altruistic and biospheric. An example of a message framed according to a hedonic value is: “Switching off 3 electrical appliances while not in use instead of putting them on stand-by will save you roughly enough money per year to go out for two restaurant meals”. The two remaining messages served as controls: one of them represented a more or less conventional way of communicating energy information using scientific units, while the other one expressed energy in terms of energy during physical exercising without scientific units. The order of presentation of messages versus values questionnaire and the order of messages were randomised among the participants to avoid carry-over and primacy or recency effects. The six messages presented to the participants are listed below (the notes in the square brackets indicate internal values framed by the messages, this information was not presented to the participants):

1. *Switching off 3 electrical appliances while not in use instead of putting them on stand-by will contribute to preservation of our planet for future generations*. **[altruistic value]**
2. *Switching off 3 electrical appliances while not in use instead of putting them on stand-by will protect the Earth from harmful climate change*. **[biospheric value]**
3. *Switching off 3 electrical appliances while not in use instead of putting them on stand-by will save roughly 60 watts of electrical energy per year*. **[control message]**
4. *Switching off 3 electrical appliances while not in use instead of putting them on stand-by will save you roughly enough money per year to go out for two restaurant meals*. **[hedonic value]**
5. *Switching off 3 electrical appliances while not in use instead of putting them on stand-by will make you better off by roughly 40 a year*. **[egoistic value]**
6. *Switching off 3 electrical appliances while not in use instead of putting them on stand-by will definitively save roughly the same amount of energy per year as it takes to cycle up a steep hill for 10 min*. **[control message]**

**Table 2:** Correlation matrix of the four internal values and six energy feedback messages

Message	Altruistic	Biospheric	Egoistic	Hedonic
Scientific units	0.03	0.35	0.28	0.11
Physical exercise	0.23	0.19	0.40*	0.08
Altruistic message	0.16	0.61**	0.03	0.18
Biospheric message	0.27	0.68**	0.09	0.12
Egoistic message	0.02	0.48**	0.12	0.32
Hedonic message	0.10	0.50**	$-$0.05	0.15

* Correlation is significant at 0.05 level (2-tailed)

** Correlation is significant at 0.01 level (2-tailed)

**Results** Pearson product-moment correlations were performed on the six messages and the four environmental values. The results of the analysis are shown in Table 2. There are significant high correlations between biospheric values and all value-framed messages. Significant correlations are also found between egoistic value and the control message on energy information expressed in terms of physical exercise: *r*(28) = 0.4, *p* < 0.05.

**Discussion** Correlation results found in this analysis should be interpreted with care, as the main limitation of the current study is the low number of energy-framed messages. We used only 6 messages in order not to burden the participants and there were only 30 participants. Another limitation is the face validity of messages: they do not always represent the value they are supposed to, because of conceptual overlap between altruistic and biospheric formulations. Further, not all messages are of equal length; some of them convey very specific information, while other cannot, due to the impossibility of accurate calculation of such information (e.g. environmental impact from an altruistic point of view).

Our hypothesis was that the appeal and persuasive power of value-framed messages would be highly correlated with respective values. This hypothesis is only partially supported by the analysis results. It appears that biospheric value is highly correlated with all value-framed messages and not correlated with the control messages. It suggests that people with low biospheric values would not prefer value-framed energy messages and people with high biospheric values would find any value-framed energy information appealing. On the contrary, people with high egoistic or financial values would prefer accurate energy information in physical exercise terms. However, when only one question—relating to an egoistic value that assesses a financial component—is analysed, it results in a significant correlation between egoistic values and an egoistically-framed message. The results might be explained by one more hidden variable: trust. One of the participants said that she did not like the messages about energy savings per year in terms of money or number of restaurant meals (egoistic and hedonic messages respectively) because she thought that the information was not true. In contrast, messages in scientific units and physical exercising might sound more trustworthy and therefore people with high egoistic values might prefer them more. The result that no correlations were found between altruistic messages and altruistic values can be explained by the low discriminant validity of an altruistic energy information message, due to the fact that the formulation of the message is potentially ambiguous and might emphasise biospheric more than altruistic values.

Based on the results of the study, we can also conclude that the persuasive potential of internal values depends on initial personal predispositions towards energy conservation (high biospheric values) which implies that only individuals with high biospheric values will be sensitive to persuasive technologies based on internal values.

### Study 2: Sensitivity analysis

For a purely explorative purpose, model sensitivity analysis was performed in order to understand the relationships between input and output variables in the computational model, to test model’s robustness and to investigate the inputs to which the model’s output is most susceptible—in the light of the previous empirical validation study. By changing all the model inputs gradually within the possible values range which corresponds to [0,1] interval according to models assumptions, it was found that the most sensitive inputs into the model are Energy literacy, Trigger/cue and Success expectancy. Assigning 11 different values to Energy literacy within [0,1] range starting from a minimal value of zero with an increment of 0.1 resulted in 24% output value change (see Table 3). For Success expectancy and cue it was 20%. The least sensitive input is each of personal values (altruistic, hedonic, biospheric, egoistic) which resulted in only 5% output value change. We adopted one-factor-at-a-time approach which suggests changing one input variable and keeping others at their baseline value.

Model parameters were also changed gradually to observe the effect in parameter value changes. This analysis reveals that the most sensitive parameters are the weights between motivation and ability and behavioural intention which led to a 46% change in the model output.

**Table 3:** Model output value increase as a function of inputs changes within [0,1] range and an increment of 0.1

Input variable	Output value increase (%)
Success expectancy	20
Barriers	13
Energy literacy	24
Altruistic value	5
Hedonic value	5
Egoistic value	5
Biospheric value	5
Cue (one component)	20

The results of the sensitivity analysis are in line with the results of an empirical evaluation in Study 1 which suggested that tailoring to personal values would not be persuasive for all individuals.

### Study 3: Model prediction of users’ energy consumption state prior to persuasive technology

We perform predictive validation of the model using the electricity consumption data from 20 households. For this evaluation stage, a modified version of the model is used which includes only the energy behaviour part of the model without an intervention cue effect at the level of behavioural intention. The purpose of this validation is to evaluate whether a model with static variables and without any behaviour change interventions can predict users energy consumption behaviour.

**Procedure** Survey data on personal values, success expectancy, barriers and energy literacy was obtained from 20 households which are part of the ENLITEN project.^1^ The cognitive component of energy literacy was assessed with 7 multiple choice items from the British Energy Literacy survey (Cotton et al. 2015). Personal values were assessed with a 16-item 5-point Likert-scale questionnaire adapted from Groot and Steg (2008) and Steg et al. (2014a). Success expectancy was measured by 3 items taken from a survey which was especially designed for the ENLITEN project. The items were 5-point Likert scale statements. The same holds for the assessment of perceived barriers. The paper surveys were administered to each household and either returned by post, or returned on the day of administration. Five households of the sample were single person households, while the rest were households consisting of more than one person. In all households the person who filled in the survey was the one who was responsible for paying energy bills. Another unpublished study in the context of the ENLITEN project demonstrated that there is a correlation between personal values of a bill payer and energy consumption of the bill payer’s household even if there are more than one person in the household. This justifies our usage of bill payers’ survey data as a good representation of households’ energy consumption determinants. The survey data was converted to a scale from 0 to 1 in order to produce the inputs according to the model format. The electricity consumption data was obtained from electricity sensors, in which the energy data was expressed in kWh for a 3-week period in December 2015. For each household, an average electricity consumption over the 3 weeks was calculated, normalised with respect to other households and mapped to [0,1], then expressed in terms of energy saving behaviour $V_{beh}$ (see Eq. 8 in Sect. 4) which in this context of model validation was equal to a difference between maximum electricity consumption and observed electricity consumption:

9 $$\begin{aligned} V_{beh} = max\_consumption - observed\_consumption \end{aligned}$$

Since all consumption values are normalised to [0,1], the maximum consumption value is 1 and energy saving behaviour $V_{beh}$ is equal to 1—$observed\_consumption$.

Furthermore, the model inputs were instantiated with the survey data on personal values, success expectancy, barriers and energy literacy. 20 simulations with the real input data were performed and the output of each simulation was compared to the actual electricity consumption data for each household. An absolute error between the model output and the real data was calculated. 5 households (25% of the data) were randomly selected for a model calibration: it was noticed during the comparison of model outputs and the electricity consumption data that the energy literacy variable is more associated with energy consumption than perceived barriers. It was decided to adjust the initial parameter values which correspond to the connections of energy literacy and barriers with ability: the energy literacy concept was given more influence by changing its weight $w_{lit}$ from 0.4 to to 0.5 and the barrier weight $w_{bar}$ was changed from 0.4 to 0.3 correspondingly.

Sensitivity analysis indicates that weights related to motivation and ability have a large effect on behavioural intention. In line with this and the social practices theory which states that our behaviour is more determined by the environment rather than by individual motivations, an alternative model with modified weights related to motivation and ability was also tested. In this alternative model, the contribution of ability was higher than that of a motivation in line with the social practices theory: the alternative model had weights of 0.6 and 0.4 for ability and motivation, respectively, as against 0.5 and 0.5 in the initial model.

Finally, a further 20 simulations of the same model with random inputs were performed and absolute error between the model output and the real data calculated.

**Results** The average error for the random inputs is 0.4, while the average error of the original model is 0.265 and the error of the model with modified parameters is 0.260. One-way Repeated Measures Analysis of Variance (ANOVA) was performed to investigate the significance of the difference between the three models. The statistical analysis results indicated a significant model effect, $F(2, 38) = 14.58$, $p<.01$, effect size $Partial \;\eta ^2 = 0.43$. We conducted two pairwise comparisons among the means for the model with equal contributions of motivation and ability (Model 1), the model with a higher ability contribution (Model 2) and the random model with parameters set as in Model 1 (Random Model). We compared Model 1 with the Random Model, Model 2 with the Random Model and Model 1 with Model  2 using Holm’s sequential Bonferroni procedure. The results indicate that comparisons of Model  1 with Random model and Model 2 with Random model were found significant while the comparison of Model  1 and Model 2 was not found significant. This analysis indicates that both models with real inputs perform significantly better than a model with random inputs, although Model 2 was not better than Model 1.

### Study 4: Model predictions of the effect of persuasive technology

In this study we perform a validation of model predictions regarding the effect of persuasive technology. In the context of the given model, we investigate the influence of a digital behaviour change cue on energy related behaviour. For this evaluation we used the same sample of ENLITEN households as for Study three (see Sect. 5.3). In these households a digital energy feedback system was deployed for a period of 3 months (January 2016–March 2016). The system was an energy feedback application called iBert which was presented via a tablet computer. The application provided different types of energy feedback within a counterbalanced within-subject experiment. The main goals of the experiment and the design of the application are not the main focus of the current evaluation, more details and results of the ENLITEN project study on energy behaviour change are presented in Mogles et al. (2017). Only the electricity consumption data obtained from this field experiment are used to evaluate the current behaviour change model.

**Procedure and materials** Energy feedback was presented via a tablet computer, a Kindle Fire 7 inch touchscreen display, dimensions 3 $\times$ 27 $\times$ 17 cm, IPS LCD screen type, display resolution: 1024 $\times$ 600, 1.2 GHz processor, 1 GB RAM. The tablets were deployed in the second half of December 2015, though the application was not activated until the 3rd day of January 2016 when the behaviour change interventions study began. Each household received four types of digital energy feedback:

A. Standard feedback in kWh and financial costs on daily and weekly electricity and gas consumption along with in-home environment variables such as home temperature, humidity, CO_2_ level; in the context of the current energy behaviour change model, this feedback corresponds to a cue which communicates live status of energy consumption and triggers egoistic value, given the information in financial terms (context information and Egoistic components activation in Fig. 2). In the context of design principles of persuasive technologies proposed by Oinas-Kukkonen and Harjumaa (2008), this type of a display deploys only an information tailoring strategy (adjusts information to users context).
B. Standard feedback with internal values where the information in kWh was translated into one of the three internal values associated with pro-environmental behaviour: altruistic, biospheric and egoistic (value selection was not tailored, but randomly selected). It was decided not to translate the feedback according to the hedonic value due to some difficulties with the operationalisation of this value revealed during two small pilot studies; according to the current model, feedback of type B represents a cue with an activation of Context information, Egoistic value components and one of the three internal values components—Biospheric, Egoistic or Altruistic (see Fig. 2). This type of a display deploys the information tailoring strategy from persuasive technologies, enhanced by a generic internal value approach from environmental psychology (translates energy usage context information into one of the three mentioned earlier internal values which are related to pro-environmental behaviour).
C. Standard feedback with informational messages on energy related behaviour with action prompts tailored to the household’s building characteristics; type C feedback represents a cue that contains textual messages with action prompts influencing Context information, Egoistic value, Energy literacy and Behavioural intention components of the model. This type of energy feedback deploys reduction (reduces energy consumption to usage of electrical appliances), tunneling (provides clear action prompts) and information tailoring strategies.
D. Standard feedback with informational messages on energy related behaviour tailored to the household’s building characteristics. This display combines features of displays type B and C: informational messages on energy related behaviour with action prompts tailored to the household’s building characteristics, and translated according to one of the three internal values. The cue/trigger in Type D feedback influences Context information, Egoistic value, Energy literacy and Behavioural intention components along with of the three internal values—Biospheric, Egoistic or Altruistic in the context of the proposed model. This type of energy feedback deploys reduction (reduces energy consumption to usage of electrical appliances), tunneling (provides clear action prompts) and tailoring strategies enhanced by a generic internal value approach from environmental psychology.

The textual messages in type C and D feedback types were comparable in terms of length and content, messages of display B were shorter because they did not tailor information to users’ buildings. Feedback of type A provided only basic live status of energy consumption and some environmental variables such as home temperature and $\hbox {CO}_2$ level. Each energy feedback type lasted 3 weeks. Digital interventions targetted four types of energy related behaviour: home internal temperature, heating schedule, ventilation level and electricity consumption. For the current model evaluation we focus only on electricity consumption. Households are randomly assigned to one of the four feedback types according to a Latin square counterbalancing design.

Messages in feedback types C and D are sent only if there are energy wasting events identified by intelligent algorithms. Thus, if no energy wasting events are identified within a household, the household would receive only energy feedback types A and B. An electricity consumption message addresses high electricity consumption during periods when no one is at home. An electricity related message of type C is as follows: “I have noticed that your electricity use was higher than expected last week, during periods when no one might have been home. This consumes energy unnecessarily. Repeated regularly, your home might waste up to $\ldots$ kWh of energy over a whole year. Advice: Write a note reminding yourself to turn off the lights, TV, computers and other electronic devices when you leave home.” An example message of type D with energy information translated according to egoistic value (financial costs) is as follows: “I have noticed that your electricity use was higher than expected last week, during periods when no one might have been home. This consumes energy unnecessarily. Repeated regularly, this might cost you ...more over a whole year. Advice: Write a note reminding yourself to turn off the lights, TV, computers and other electronic devices when you leave home”.

For the model dynamics evaluation, we measured daily average electricity consumption before and after the digital interventions in the same sample of 20 households used for the static component of model evaluation described previously in Sect. 5.3. However, in contrast to the previous evaluation, we took a daily average consumption within a 3-week period in from mid December 2015 instead of a 3-week average consumption during the period of end November-first half December 2015. For the post-intervention period, we took the daily average electricity consumption data for the same homes during the 3-week period after the 3-month digital interventions. The post-study electricity consumption measurement come from April 2015. The electricity data in financial terms is normalised and mapped to [0,1]. The actual difference between the electricity consumption before and after the interventions was compared to the simulated consumption difference before versus after the interventions. The model inputs for success expectancy, internal values, barriers and energy literacy were the same values as in Study 3, derived from the surveys described in Sect. 5.3. The survey values were normalised and mapped to [0,1]. Given the fact that all homes received all four types of cue, although in different orders, for the model simulation one universal cue was used as an input. This cue vector contained all 4 types of actual interventions along with the no-cue period prior to the interventions and corresponds to the following energy feedback type dynamic sequence: [No cue, cue of type B, cue of type C, cue of type D, cue of type A]. Each item in this sequence represents a 3-week period. We assume that one time step in the model corresponds to 1 week, given the nature of behaviour change interventions and the frequency of tailored messages, which were updated once a week.

Due to the absence of some electricity consumption data for five households, these homes were excluded from the analysis which left us with only 15 homes having all model input and output data during the periods before and after the interventions.

**Results** A paired sample *t* test was conducted to investigate if there was any significant effect of digital interventions on electricity consumption. Results indicate that the mean daily energy consumption [expressed in financial terms—UK pounds (£)] before the digital interventions ($M = 1.8$, $SD = 1$) is not significantly higher than the mean daily electrical energy consumption after the interventions ($M = 1.6$, $SD = 1$), $t(14) = 1.1$, $p = 0.29$.

Six homes within the sample did not receive any messages with display types C and D because no electrical energy wasting events were identified within these homes. Nine homes did receive tailored messages. An actual observed difference in electricity usage behaviour between the period before the interventions and after the digital interventions for homes with and without tailored messages is shown in Table 4.

**Table 4:** Observed energy consumption behaviour difference for 15 households on [0,1] scale before versus after the digital interventions

	Messages	No messages
	$-$ 0.04	0.12
	$-$ 0.12	− 0.01
	$-$ 0.01	0
	0.01	0.01
	$-$ 0.17	0.01
	0.06	0.06
	0.66	No data
	0.07	No data
	0.13	No data
Total average	0.07	0.03

A positive value indicates a positive effect of persuasion. Each cell denotes the ‘before-after persuasion’ consumption difference for one household

A negative value in Table 4 means a negative difference between energy consumption behaviour before and after the interventions, which indicates a negative effect from an intervention. A positive value indicates that a household used less electrical energy after the interventions and a positive effect of an intervention. As it can be seen in Table 4, 9 homes of 15 increased their energy consumption after the interventions and one home did not show any change. For the homes which received tailored messages with action prompts, an average difference in consumption between the period before and after the interventions was 0.07 which corresponds to a decrease in energy consumption by roughly 7% according to the [0,1] scale where 1 stands for 100%. For the homes which did not receive any tailored messages, an average decrease in energy consumption was 0.03 which roughly corresponds to 3% decrease in energy consumption over 18 weeks. An independent sample *t* test was conducted to evaluate if this difference between the groups (tailored message versus no tailored message) was statistically significant. The results indicated that the mean energy consumption change scores for households which received tailored messages ($M = 0.07$, $SD = 0.08$) was not significantly different from the scores of households that did not receive the tailored messages ($M = 0.03$, $SD = 0.02$), $t(8.98) = -\,.42$, $p = 0.69$.

An observed average decrease in energy consumption across all 15 households is 0.05 on [0,1] scale which corresponds to 5% consumption decrease. Model simulations for 15 households with the model inputs obtained from the surveys and actual digital interventions also demonstrated an average decrease of 5% in electrical energy consumption after the 18 weeks period covered by the equivalent period before, during and after the interventions, with a minimal decrease of 2% and a maximal decrease of 6%. These results demonstrate than the behaviour change speed simulated by the model perfectly fits the dynamics observed in our sample of households. Given this fit of the model’s dynamics behaviour to the real data, no changes to the initially chosen parameters for dynamics were made.

Regarding the effect of persuasive technology based on the model, there was no significant effect of persuasive technology. This can be explained by a small sample size in combination with a rather moderate effect of such technologies a priori, given the range of effects reported in literature (Barbu et al. 2013; Abrahamse et al. 2007). The majority of applications for energy behaviour change, including the one presented in the current study, do not deploy many persuasive strategies proposed in literature (Oinas-Kukkonen and Harjumaa 2008). They do not fully utilise *personalisation* strategy as well which is considered of the most promising persuasion strategy that meeds more exploration (Orji 2014). It concerns the current study as well: we applied generic strategies for persuasion based on internal values, energy literacy and action prompts along with reduction, tunneling and information tailoring. In spite of the fact that the sample is quite small, it provides some indications regarding the moderate expected effect of given behaviour change interventions over time.

Given the complex nature of energy consumption behaviour (also grasped by our model), persuasive technologies could benefit from more personalisation regarding users’ goals, needs, mental models and personal characteristics. The current model still can be used to design better personalised persuasive systems which can address personal Success expectancy, Barriers (habits, perception of social norms and practices), level of Energy literacy.

The four model evaluation studies reveal that the model is capable of reasonable prediction of typical energy saving behaviour of households without persuasive technologies (Study 3) and that the model is capable of prediction of dynamic changes in energy saving behaviour as a function of persuasive technologies based on reduction, tunneling and information tailoring to users’ context strategies enhanced by a generic internal value enhancement approach (Study 4). The model’s assumption for providing personalised feedback according to internal values gained only partial support: addressing personal values seems to have an effect only on individuals with high biospheric values (Study 1). This is also in line with the model sensitivity analysis which demonstrated that personal values had the least immediate effect on energy saving behaviour and energy literacy had most effect (Study 2).

## Model simulations

In this section several model simulations are described for the purposes of demonstrating the effect of three persuasion strategies—tailoring, personalisation, generic non-tailored persuasion, tunneling—on a user over a longer period of time—over 50 weeks (approximately 1 year). Simulations are important here since it is difficult to explore the long-term effects of persuasive technologies, given the practical difficulties of performing long term longitudinal studies in this domain.

One time step in the simulations corresponds to 1 week. The model parameters used for these simulations are listed in Table 1—except of the weights from Energy literacy, Barriers and Context information to Ability/Resources which were changed after the model evaluation: $w_{bar} = 0.3, w_{lit} = 0.5, w_{context} = 0.2$ . After the model evaluation results described in Sect. 5.3 it was decided to assign more influence to energy literacy in comparison to barriers.

**Fig. 3:**
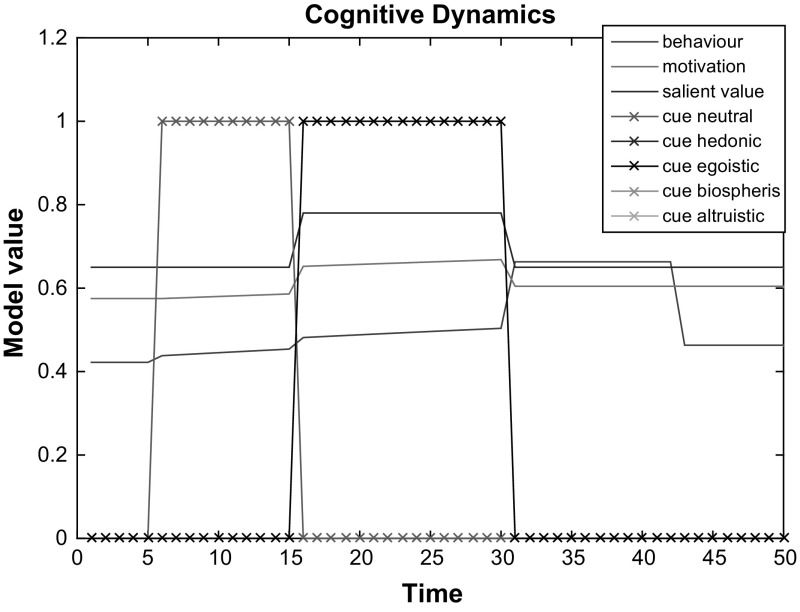
Dynamics of behaviour change as a function of persuasion for scenario 1: value-framed cues tailored to user values

### Simulation 1: Value-framed cue tailored to user values

This simulation demonstrates (see Fig. 3) dynamics of user’s behaviour as a function of the following persuasive strategies: no persuasion—information tailoring—value-based personalisation—tunneling—no persuasion.

In the context of the current model it can be described in the following way: initially during the first four time steps there are no cues (no persuasive technologies deployed) and the baseline behaviour value is quite low. From time point 5 a value-neutral cue is introduced (it can be a daily or a weekly energy feedback in kWhs or a text message providing some information on energy usage—information tailoring strategy): this results in a slight increase in a behaviour and an almost imperceptible change in motivation. From time point 16 a value-framed cue replaces the value-neutral cue (personalisation); the personal value that is addressed here is egoistic and is it tailored to the user’s personal values profile. An example of such a cue can be energy information translated into financial costs. Figure 3 shows a substantial increase in motivation and behaviour. From time point 32 the value-framed cue is eliminated and is replaced by a cue that provides just actionable prompts to a user (not visible in the Figure)—tunneling strategy, without any explanation why those actions should be taken. This cue results in decreased motivation, although its effect on behaviour is quite large; as a result behaviour increases above an average value of 0.5. At the end, from time point 44, persuasive technology is removed again (no action prompts or any other cues are introduced); during this time interval, behaviour of the user drops back to a level slightly higher than the baseline. As it can be seen here, behaviour is increased even after the removal of persuasive technologies due to the long-term effect of tailoring and personalisation. An immediate great effect of tunneling is observed here, though it is less effective in the long run because after the removal of persuasion, tunneling behaviour returns to its initial level before the tunneling strategy.

### Simulation 2: Value-framed cue not tailored to user values

**Fig. 4:**
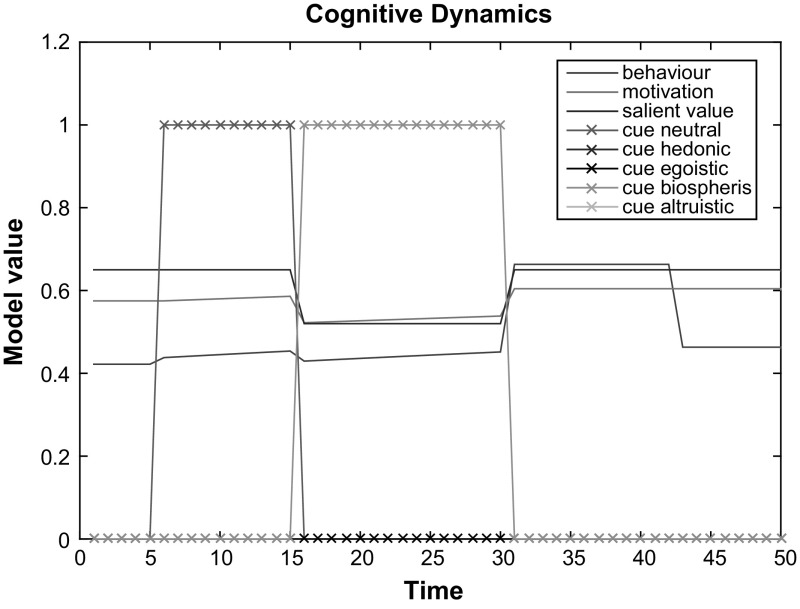
Dynamics of behaviour change as a function of persuasion for scenario 2: value-framed cues not tailored to user values

In this scenario (see Fig. 4), the setting is the same as in the previous one, except that the value-framed intervention introduced at time point 16 is *not* tailored to a user’s value profile. The intervention pattern is the following: no persuasion—information tailoring—value-based non-tailored intervention—tunneling—no persuasion. Here energy information is translated into a biospheric value, which is not high for this individual. Figure 4 shows that the motivation of this person drops during a generic value-based intervention that is not tailored to the individual’s values. As a result behaviour slightly decreases, but then gradually rises again due to action prompts which are part of tunneling strategy. It can be observed in this scenario that tailoring is more effective than generic persuasion.

### Simulation 3: Value-framed cue with action prompts

**Fig. 5:**
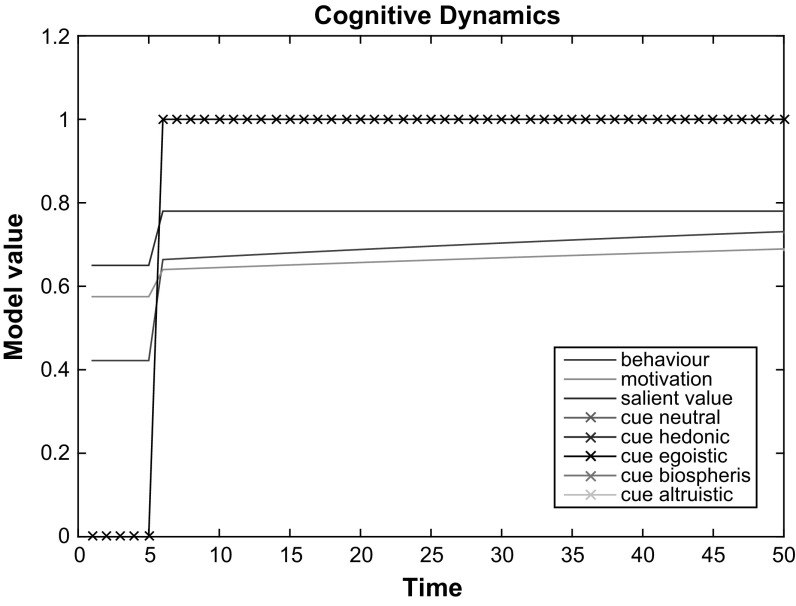
Dynamics of behaviour change as a function of persuasion for scenario 3: value-framed cues with actionable prompts

This simulation contains the following persuasion pattern: no persuasion—information tailoring combined with value-based personalisation and tunneling. In the context of the proposed model it is a combination of tailored value-framed messages and actionable prompts (see Fig. 5). This shows that after the introduction of a value-framed tailored cue with action prompts, behaviour and motivation rapidly rise and continue to increase gradually until the end of the simulation. This simulation demonstrates a joint effect of multiple persuasion strategies.

The simulation scenarios described in this section demonstrate user behaviour dynamics as a result of different behaviour change interventions with the help of persuasive technology. The model demonstrates a longer term effect of tailored to a user interventions, value-based personalisation and a short-term facilitating effect of tunneling (action prompts in the context of the given model).

## Discussion and conclusions

Energy consumption behaviour has been studied from a variety of perspectives in different disciplines and to date, there is no unified comprehensive framework for energy consumption behaviour change interventions (Burger et al. 2015; Karatasou et al. 2014). Indeed, this behaviour is too complex to approach it from one single perspective, as it involves multiple factors and determinants at both societal and individual level.

The agent-based computational model for energy behaviour change introduced in this paper takes a broad perspective on energy behaviour and behaviour change intervention, integrating theories and models from environmental and educational psychology, environmental education, sociology, HCI and persuasive technologies. The current model is designed to demonstrate mechanisms behind persuasive technologies for energy consumption behaviour. Most energy behaviour interventions in the energy domain take an ‘instrument’ approach to changing behaviour, without explicit consideration of behaviour determinants (Karatasou et al. 2014). The authors of this recent review emphasise that “focus only on the instrument of an intervention, without an analysis of energy related targeted behaviours and their behavioural antecedents masks the real potential of energy savings and fails to rigorously assess the true impact of the instrument in question”. The model presented here addresses this gap in digital energy feedback and interventions research.

The static component of the model (energy consumption behaviour without persuasive technologies) and the dynamic behaviour change interventions component (behaviour with persuasive technologies) are validated using survey data and electricity consumption data from a number of households within the ENLITEN project. The validation of the models’ behaviour without interventions demonstrates that the model can predict energy-related behaviour much better than a model with random inputs. The validation of an effect of persuasive technologies based on tailoring, tunneling and reduction principles (Oinas-Kukkonen and Harjumaa 2008), reveals that the 5% improvement in behavioural intention (simulated in the model), as a result of digital behaviour change interventions, corresponds to an average behaviour improvement observed in real data. It is also in line with the 7–20% savings effect as a result of digital energy feedback reported in literature (Darby 2010; Hargreaves et al. 2010; Vine et al. 2013). However, the data revealed a substantial variability across the households’ energy consumption behaviour and for intelligent behaviour change applications the parameters of the model should be calibrated against individual households which was not done in the current work.

In light of the hypothesis regarding the effect of interventions that are tailored to personal values, defined in Sect. 5, the model simulations suggest that tailored value-framed cues initially have a smaller effect than action prompts, but they induce long-term behaviour change after the elimination of the cues, while action prompts have a larger effect, but this effect disappears after the elimination of these cues.

The main limitations of the current study are: (i) the sample size is small with only 20 households for the static component validation and 15 households for the dynamic interventions; given this sample size and an expected effect of persuasive technologies in energy consumption domain, it is difficult to draw any convincing conclusions about model’s correctness; (ii) only electricity consumption was taken for the validation of the model which is just a generic proxy for concrete energy related actions and behaviours which are difficult to track; (iii) only three persuasive strategies out of 28 proposed in Oinas-Kukkonen and Harjumaa (2008) were applied during model evaluation studies and personalisation strategy was not implemented which could diminish the persuasive potential of interventions; (iv) long-term effect of the interventions needs to be evaluated in a longitudinal and large-scale study in order to be able to claim effects. Nevertheless, the work presented here is an encouraging step towards an holistic personalised agent-based model for intelligent behaviour change interventions.

The model we present can provide a suitable framework for persuasive technologies, as it integrates different overlapping theories and approaches from several disciplines. The current model can be directly embedded in a multi-agent support system for an intelligent personalised energy feedback and behaviour change interventions and then used by a reasoning component in a software agent to tailor energy feedback to users needs and cognitive profiles. Further, the model can be expanded into a multi-agent model to explore energy policy effects at population level. In these ways, it can inform designers and engineers of digital energy feedback systems, as well as the policy makers who make decisions and regulations regarding digital energy feedback.

We summarise the main contributions of the paper by discipline as follows:

- For HCI: informs HCI designers, introducing formal modelling and simulations for an intelligent design of complex behaviour, offering a tool for persuasive technologies that brings transparency and insights into the mechanisms of behaviour change over time;
- For AI: assists the design of intelligent agents for energy feedback and behaviour change interventions;
- For the energy domain: integrates different theories of behaviour change at the level of individual actors for an intelligent energy feedback;
- For psychology: formalises and simulates value-framing and goal-framing theories of environmental behaviour.

In the future, a larger sample of households should be used for model validation. The model can also undergo a more precise validation process against actual specific behaviour of individuals or households as a result of model-based tailored interventions. If these future studies demonstrate the model’s appropriateness, the model might be utilised by persuasive technologies within a user tailored system for energy consumption behaviour change. Machine learning algorithms might also be used in order to adjust model parameters to specific users and to enhance the effect of persuasive technology of behaviour change by means of personalisation. In its current state, the model can be used for a simulation of long term effects of different behaviour change strategies within persuasive technologies since long term field experiments with such technologies are difficult to sustain.

## Footnotes

1
http://www.cs.bath.ac.uk/enliten/.
